# Arterial pressure-based cardiac output in septic patients: different accuracy of pulse contour and uncalibrated pressure waveform devices

**DOI:** 10.1186/cc9058

**Published:** 2010-06-10

**Authors:** Xavier Monnet, Nadia Anguel, Brice Naudin, Julien Jabot, Christian Richard, Jean-Louis Teboul

**Affiliations:** 1 AP-HP, Hôpital de Bicêtre, Service de Réanimation Médicale, 78, Rue du Général Leclerc, Le Kremlin-Bicêtre F-94270, France; 2 Université Paris-Sud 11, Faculté de Médecine Paris-Sud, EA 4046, Le Kremlin-Bicêtre, 63, rue Gabriel Péri F-94270, France

## Abstract

**Introduction:**

We compared the ability of two devices estimating cardiac output from arterial pressure-curve analysis to track the changes in cardiac output measured with transpulmonary thermodilution induced by volume expansion and norepinephrine in sepsis patients.

**Methods:**

In 80 patients with septic circulatory failure, we administered volume expansion (40 patients) or introduced/increased norepinephrine (40 patients). We measured the pulse contour-derived cardiac index (CI) provided by the PiCCO device (CIpc), the arterial pressure waveform-derived CI provided by the Vigileo device (CIpw), and the transpulmonary thermodilution CI (CItd) before and after therapeutic interventions.

**Results:**

The changes in CIpc accurately tracked the changes in CItd induced by volume expansion (bias, -0.20 ± 0.63 L/min/m^2^) as well as by norepinephrine (bias, -0.05 ± 0.74 L/min/m^2^). The changes in CIpc accurately detected an increase in CItd ≥ 15% induced by volume expansion and norepinephrine introduction/increase (area under ROC curves, 0.878 (0.736 to 0.960) and 0.924 (0.795 to 0.983), respectively; *P *< 0.05 versus 0.500 for both). The changes in CIpw were less reliable for tracking the volume-induced changes in CItd (bias, -0.23 ± 0.95 L/min/m^2^) and norepinephrine-induced changes in CItd (bias, -0.01 ± 1.75 L/min/m^2^). The changes in CIpw were unable to detect an increase in CItd ≥ 15% induced by volume expansion and norepinephrine introduction/increase (area under ROC curves, 0.564 (0.398 to 0.720) and 0.541 (0.377 to 0.700, respectively, both not significantly different from versus 0.500).

**Conclusions:**

The CIpc was reliable and accurate for assessing the CI changes induced by volume expansion and norepinephrine. By contrast, the CIpw poorly tracked the trends in CI induced by those therapeutic interventions.

## Introduction

Cardiac output is regarded as one of the most important variables to be monitored in patients with acute circulatory failure [1]. PiCCO and Vigileo devices are two commercially available monitors that estimate cardiac output by analyzing the arterial pressure waveform. These techniques are attractive because they provide a ready and continuous measure of cardiac output and because they do not require intracardiac catheterization [2]. Arterial pressure waveform analysis is based on the physiological principle that stroke volume is physiologically related to the arterial pressure wave and aortic impedance.

Nevertheless, these two techniques differ in the way they estimate aortic impedance. The PiCCO device provides an estimation of the cardiac index from an analysis of the pulse contour (CIpc), but it does not estimate aortic impedance because it calibrates CIpc from a measurement of cardiac index obtained from transpulmonary thermodilution (CItd), a technique that has demonstrated a robust accuracy in comparison with pulmonary artery thermodilution [3-9]. In contrast, the Vigileo device provides an estimation of cardiac index from an analysis of the pulse waveform (CIpw), but it does not require any external calibration because it estimates aortic impedance from certain characteristics of the arterial pressure waveform and from some demographic data [10]. Some studies in cardiac surgery patients suggested that CIpw reliably measures cardiac index when compared with the pulmonary artery catheter-derived measurement [11-15]. However, the reliability of the CIpw has been questioned [16-21]. In particular, it has been suggested that the uncalibrated pressure-waveform analysis would not reliably track the changes in cardiac output when the arterial tone changes to a large extent [20] or during hyperdynamic states [22-24]. This raises the question of the suitability of CIpw for critically ill patients with hemodynamic instability. In particular, the device could perform differently if the changes in cardiac output were related to volume expansion or to vasopressor administration during septic shock.

Therefore, we assessed the respective abilities of CIpc and CIpw to track the changes in CItd induced by volume expansion or by norepinephrine in sepsis patients.

## Materials and methods

### Patients

After approval by our Institutional Review Board (Comité pour la Protection des Personnes île-de-France VII), we enrolled 80 patients who had circulatory failure of septic origin in our medical intensive care unit. Acute circulatory failure was defined by the presence of one or more of the following signs: (i) systolic arterial pressure ≤90 mm Hg (or decrease in systolic arterial pressure ≥50 mm Hg in known hypertensive patients); (ii) urinary flow ≤0.5 ml/kg/hr for more than 2 hours; (iii) tachycardia ≥100 beats/min; and (iv) skin mottling [25,26]. Patients' relatives were informed about the study at the time the patient was included. They were given a choice to refuse the patient's participation at that time. If not, patients were informed as soon as their mental status allowed, and they were given the choice to withdraw their participation in the study.

In 40 of these patients (Group 1), the attending physician decided to administer fluid because the patient showed some criteria predicting fluid responsiveness: increase in cardiac index (CI) ≥10% during a passive leg-raising test [27]; increase in cardiac index (CI) ≥5% during an end-expiratory occlusion [28]; or, in cases with no spontaneous triggering of the ventilator and no cardiac arrhythmias, a pulse-pressure variation ≥13% [29]. In 40 different patients (Group 2), the attending physician had decided to introduce norepinephrine or to increase its dosage because the mean arterial pressure was <65 mm Hg (or 75 mm Hg in known hypertensive patients) [30] and the diastolic arterial pressure was low [31].

All patients had a catheter inserted into the internal jugular vein and a catheter inserted into the femoral artery (Pulsiocath for thermodilution; Pulsion Medical Systems, Munich, Germany). The arterial line was divided in two branches, one connected to a PiCCOplus device (PiCCOplus v6.0; Pulsion Medical Systems, Munich, Germany), and the other one connected to a FloTrac/Vigileo device (FloTrac/Vigileo v1.10; Edwards Lifesciences, Irvine, CA). This enabled the two devices simultaneously to analyze the same sample of the arterial pressure curve.

### Interventions and measurements

Before each therapeutic intervention (that is, before administering fluid infusion in Group 1 and before introducing/increasing norepinephrine in Group 2), we performed a first set of hemodynamic measurements, including heart rate, systemic arterial pressure, CItd, CIpc, CIpw, and systemic vascular resistance (SVR). We used the values of CIpc and CIpw that were automatically displayed on the screens of the commercial devices. The CIpc and CIpw displayed by the monitors are averaged over a 12-second and 20-second rolling period, respectively. We could not obtain the digital (unprocessed, unfiltered, and nonaveraged) data from the devices. In practice, the CIpc and CIpw values displayed by the monitors were averaged over 10-second periods. The CItd was measured by the PiCCOPlus device by injecting 15 ml of iced saline (<8°C) through the central venous line. The injection was manually performed in triplicate, and the values of CItd were averaged. Immediately after performing thermodilution boluses, the values of CIpc were measured, the arterial line being physically shared in Y through taps toward the Vigileo and the PiCCO devices. Because the thermodilution automatically calibrated the pulse-contour analysis of the PiCCO device, CIpc was identical to CItd at the starting time. The CIpw was carried immediately before thermodilution to avoid interference between the temperature drift and the accuracy of the CIpw. The total SVR was estimated as mean arterial pressure divided by CItd.

After the first set of hemodynamic measurements was completed in Group 1, fluid loading was performed by infusing 500 ml of saline over a 30-minute period. In Group 2, norepinephrine was titrated, targeting a mean arterial pressure of 65 mm Hg [30] (or 75 mm Hg in previously hypertensive patients). All other treatments were kept unchanged during the therapeutic intervention. In particular, the dosage of norepinephrine was kept constant in patients of Group 1 who already received norepinephrine, and volume expansion was not administered to the patients of Group 2 during the study period.

A second set of hemodynamic measurements was carried out again after the therapeutic intervention (that is, at the end of fluid administration in Group 1 and 5 minutes after stabilization of mean arterial pressure was obtained in Group 2). This set included heart rate, systemic arterial pressure, CIpc, CIpw, CItd, and SVR. The values of CIpc and of CIpw were recorded before the CItd. Therefore, the value of CIpc was not automatically calibrated by the thermodilution. For that recording, the arterial line was shared in Y through a tap turned on to both the Vigileo and the PiCCO devices.

For 10 patients of Group 1 and 10 patients of Group 2, radial and femoral arterial catheters were simultaneously in place. In these patients, hemodynamic worsening required switching from a simple radial blood-pressure monitoring to a more complete hemodynamic monitoring like transpulmonary thermodilution.

Just after the femoral catheter insertion and before the radial catheter ablation, we took the opportunity to have both catheters in place for testing whether connecting the Vigileo device to the radial or to the femoral arterial line could provide different measures of cardiac output. For this purpose, before and after the therapeutic intervention, immediately after recording the CIpw obtained from the femoral line, the Vigileo device was disconnected from the femoral line and connected to the radial line, and the CIpw obtained from the radial line was recorded.

In these same patients, we also tested whether splitting the arterial line into two branches introduced differing harmonic influences into the system that might influence the monitors. For this purpose, before and after the therapeutic intervention, the CIpc and CIpw were recorded again after turning on the tap sharing the femoral arterial line, such that the lines directed to both devices were alternatively closed.

### Statistical analysis

All data were normally distributed (Kolmogorov-Smirnov test), except CItd, CIpc, CIpw, and the dosage of norepinephrine, and are expressed as median [25^th ^to 75^th ^percentile]. In each patient, we performed only one pair of measurements (before/after therapeutic intervention). Comparisons between values recorded before with values recorded after therapeutic interventions were performed in both groups by using a paired Student *t *test or a paired Wilcoxon test, as appropriate. Comparisons between Group 1 and Group 2 were performed with a two-tailed Student *t *test or a Mann-Whitney *U *test, as appropriate.

We compared the relative changes of CIpc and of CIpw with those of CItd during the therapeutic interventions by a Bland and Altman analysis (for absolute changes) and by linear regression analysis (for percentage changes). We tested the ability of CIpc and CIpw to detect an increase in CItd ≥ 15% by constructing receiving operating characteristics (ROC) curves. The area under the ROC curves (expressed as mean (95% confidence interval) were compared by using the Hanley-MacNeil test. This analysis was also separately performed in patients in whom the SVR changed (increased or decreased) by >15% with the therapeutic interventions and in patients in whom the SVR changed (increased or decreased) by <15% with the therapeutic interventions [32]. The values recorded before therapeutic interventions were not compared, because at this time, the CIpc was, by definition, identical to the CItd value because of calibration. After the therapeutic interventions, we compared the absolute values of CIpc and of CIpw with that of CItd by a Bland and Altman analysis and calculated the percentage error as 2 SD/mean [33].

The precision of each method was calculated from data obtained from a sample made of the first 20 and the last 20 patients included in the study when arterial pressure was stable. For CItd, we calculated the coefficient of variation (ratio of the standard deviation to the mean) for each set of three consecutive thermodilution boluses and then averaged it for the series of the 20 sets. For CIpc and CIpw, we collected the 10 consecutive values of CI displayed on the monitor. We calculated the coefficient of variation for each set and averaged it for the series of 20 sets [32].

A *P *value < 0.05 was considered significant. The statistical analysis was performed by using Statview 5.0 software (Abacus Concepts, Berkeley, CA).

## Results

### Patient characteristics

The characteristics of the patients at baseline are summarized in Table 1. All patients had a circulatory failure of septic origin. Twenty-four (60%) patients of Group 1 received norepinephrine at baseline at 0.41 (0.27 to 0.58) μg/kg/min, and this dosage was kept constant during the study period. In group 2, 29 (72%) patients received norepinephrine at baseline at 0.45 (0.12 to 0.85) μg/kg/min, and this dosage was increased to 0.64 (0.38 to 1.50) μg/kg/min. In group 2, 11 (28%) patients did not receive norepinephrine at baseline, and norepinephrine was introduced at 0.13 (0.11 to 0.21) μg/kg/min. The second set of measurements was recorded 27 (25 to 29) minutes after the first set in Group 1 and 31 (26 to 33) minutes after the first set measurement in Group 2.

**Table 1:** Patient characteristics at baseline

Age (years)	59	(53 to 72)
Gender (M/F)	37:43	
SAPS II	41	(37 to 80)
ARDS (*n*, %)	38	(47)
Source of infection		
Pneumonia (*n*, %)	64	(80)
Peritonitis (*n*, %)	6	(7)
Endocarditis (*n*, %)	5	(6)
Fasciitis (*n*, %)	2	(3)
Unknown	3	(4)
CItd (L/min/m^2^)	3.1	(2.2 to 3.5)
Vasopressors		
Norepinephrine (*n*, %)	53	(66)
Dosage of norepinephrine (μg/kg/min)	0.43	(0.21 to 0.71)
Dobutamine (*n*, %)	6	

n = 80.

Data are expressed as median [25% to 75% interquartile] or as number (%).

SAPS, Simplified Acute Physiologic Score; ARDS, acute respiratory distress syndrome; MAP, mean arterial pressure; CItd, cardiac index measured with transpulmonary thermodilution.

### Comparisons between CItd, CIpc, and CIpw in the whole population

Considering the population as a whole, the therapeutic interventions significantly increased the mean arterial pressure, CItd, CIpc, CIpw, and the SVR by 21 (5 to 38)%, 13 (6 to 22)%, 14 (4 to 20)%, 8 (0 to 18%), and 5 (-10 to 19)%, respectively.

The bias between the absolute changes in CIpc and CItd induced by therapeutic interventions was -0.07 ± 0.73 L/min/m^2^. The coefficient of correlation between the percentage changes induced by the therapeutic interventions in CIpc and in CItd was 0.73 (*P *< 0.05). After the therapeutic intervention, the bias between the absolute values of CIpc and CItd was -0.07 ± 0.36 L/min/m^2^, and the percentage error was 22%.

The bias between the absolute changes in CIpw and CItd induced by therapeutic interventions was -0.10 ± 1.40 L/min/m^2^. The coefficient of correlation between the percentage changes induced by the therapeutic interventions in CIpw and in CItd was 0.006 (*P *= 0.11). After the therapeutic intervention, the bias between the absolute values of CIpw and CItd was -0.09 ± 0.94 L/min/m^2^, and the percentage error was 61%.

### Comparisons of CIpc and CIpw with CItd in Group 1 (volume expansion)

In Group 1, the volume expansion significantly increased the mean arterial pressure, CItd, CIpc, and CIpw by 9 (1 to 20)%, 14 (7 to 24)%, 10 (4 to 16)%, and 7 (0 to 18)%, respectively. The SVR nonsignificantly decreased by -10 [0 to -16)% (Table 2).

**Table 2:** Evolution of hemodynamic parameters during therapeutic interventions

	Group 1				Group 2			
		
	Baseline		After volume expansion		Baseline		After introduction/increase of norepinephrine	
Heart rate (beats/min)	105	(82-130)	100	(89-123)	92	(82-120)	95	(79-115)
MAP (mm Hg)	69	(67-75)	76	(70-85)^a^	52	(47-58)^b^	72	(64-78)^a^
CItd (L/min/m^2^)	3.2	(2.4-3.6)	3.7	(3.1-4.1)^a^	2.9	(2.1-3.5)	2.9	(2.5-3.7)^a^
CIpc (L/min/m^2^)	3.2	(2.4-3.6)	3.4	(2.8-4.0)^a^	2.9	(2.1-3.5)	3.0	(2.4-3.6)^a^
CIpw (L/min/m^2^)	2.9	(2.6-3.2)	3.2	(2.8-3.7)^a^	2.7	(2.5-3.0)	3.0	(2.8-3.3)^a^
SVRi (dyne/sec/m^2^/cm^5^)	1,741	1,355-2,267)	1,724	1,495-1,996)	1,832	(1,323-2,675)	2,229	(1,740-2,874)^a^
GEDVi (ml/m^2^)	649	(554-715)	725	(649-803)^a^	690	(602-474)	728	(674-790)^a^

n = 40 in Group 1, and n = 40 in Group 2.

Data are expressed as median (25% to 75% interquartile). ^a^*P *< 0.05, before versus after intervention; ^b^*P *< 0.05, Group 2 versus Group 1.

MAP, mean arterial pressure; CItd, cardiac index measured by thermodilution; CIpc, pulse contour-based cardiac index measured with the PiCCO device; CIpw, arterial-pressure waveform-based cardiac index measured with the Vigileo device; SVRi, systemic vascular resistance indexed for body surface; GEDVi, global end-diastolic volume indexed for body surface.

**Figure 1:**
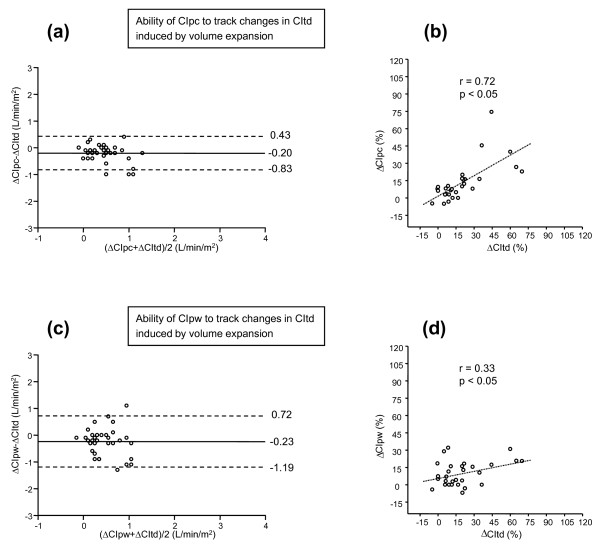
**Bland-Altman plots**. **(a, b) **Bland-Altman plot for the changes (in absolute values) and correlation (for the percentage changes) induced by *volume expansion *on cardiac index obtained by transpulmonary thermodilution (CItd) and cardiac index obtained by the pulse-contour analysis (CIpc). **(c, d) **Bland-Altman plot for the changes (in absolute values) and correlation (for the percentage changes) induced by *volume expansion *on cardiac index obtained by transpulmonary thermodilution (CItd) and cardiac index obtained by the arterial pressure waveform analysis (CIpw). Bland-Altman plots: straight line, bias; dashed line: +2 SD/-2 SD limits of agreement); dashed line, correlation line.

**Figure 2:**
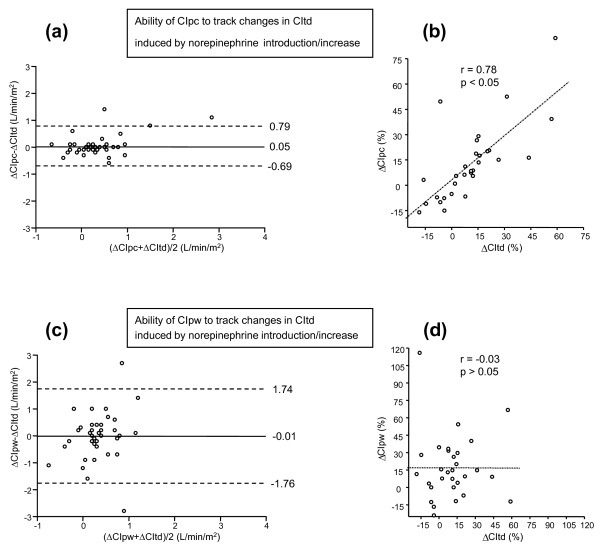
**Receiver operating characteristic (ROC) curves**. **(a) **ROC curves constructed for testing the ability of the changes in cardiac index obtained by the pulse-contour analysis (CIpc) (straight line) and of the changes in cardiac index obtained by the arterial-pressure waveform analysis (CIpw) (dashed line) to detect an increase in cardiac index obtained by transpulmonary thermodilution (CItd) ≥15% induced by *me expansion*. **(b) **ROC curves constructed for testing the ability of the changes in cardiac index obtained by the pulse-contour analysis (CIpc) (straight line) and of the changes in cardiac index obtained by the arterial-pressure waveform analysis (CIpw) (dashed line) to detect an increase in cardiac index obtained by transpulmonary thermodilution (CItd) ≥15% induced by the *introduction/increase in norepinephrine*.

The bias between the absolute changes in CIpc and CItd induced by volume expansion was -0.20 ± 0.63 L/min/m^2^. The coefficient of correlation between the fluid-induced percentage changes in CIpc and in CItd was 0.72 (*P *< 0.05) (Table 3, Figure 1). An increase in CIpc ≥ 12% detected an increase in CItd induced by volume expansion with an sensitivity of 74 (49 to 91)% and a specificity 95 (76 to 99)% (area under the ROC curve was 0.878 (0.736 to 0.960), *P *< 0.05 vs. 0.500; Table 3, Figure 2). After volume expansion, the bias between the absolute values of CIpc and CItd was -0.19 ± 0.32 L/min/m^2^, and the percentage error was 18%.

**Table 3:** Summary of the comparisons between the different techniques used for measuring cardiac index

	Changes induced by volume expansion (Group 1)	Changes induced by introduction/increase in norepinephrine (Group 2)
CIpc versus CItd		
Bland-Altman analysis for the changes in absolute value	Bias, -0.20 ± 0.63 L/min/m^2^	Bias, -0.05 ± 0.74 L/min/m^2^
Linear regression for the changes in percentage	*r *= 0.72 (*P *< 0.05)	*r *= 0.78 (*P *< 0.05)
Ability of CIpc to detect an increase in CItd ≥ 15%	Cut-off, CIpc increase ≥ 12%	Cut-off, CIpc increase ≥ 15%
	Specificity, 74 (49 to 91)%	Specificity, 93 (68 to 99)%
	Specificity, 95 (76 to 99)%	Specificity, 88 (69 to 97)%
	Area under the ROC curve, 0.878 (0.736 to 0.960)	Area under the ROC curve, 0.924 (0.795 to 0.983)

CIpw versus CItd		
Bland-Altman analysis for the changes in absolute value	Bias, -0.23 ± 0.95 L/min/m^2^	Bias, -0.01 ± 1.75 L/min/m^2^
Linear regression for the changes in percentage	*r *= 0.33 (*P *< 0.05)	*r *= -0.03 (*P *= 0.65)
Ability of CIpw to detect an increase in CItd ≥ 15%	Cut-off, CIpw increase ≥8%	Cut-off, CIpw increase ≥34%
	Sensitivity, 56 (33 to 80)%	Sensitivity, 27 (8 to 55)%
	Specificity, 71 (48 to 89)%	Specificity, 96 (80 to 99)%
	Area under the ROC curve, 0.564 (0.398 to 0.720)^a^	Area under the ROC curve, 0.541 (0.377 to 0.700)^a^

n = 40 in Group 1, and n = 40 in Group 2.

^a^*P *< 0.05 vs. CIpc.

CItd, cardiac index measured by transpulmonary thermodilution; CIpc, pulse contour-based cardiac index measured with the PiCCO device; CIpw, arterial pressure waveform-based cardiac index measured with the Vigileo device; AUC, area under the curve; ROC, receiving operating characteristics.

The bias between the absolute changes in CIpw and CItd induced by volume expansion was -0.23 ± 0.95 L/min/m^2^. The coefficient of correlation between the fluid-induced percentage changes in CIpw and in CItd was 0.33 (*P *< 0.05) (Table 3, Figure 1). An increase in CIpw ≥ 8% detected an increase in CItd induced by volume expansion, with an sensitivity of 56 (33 to 80)% and a specificity 71 (48 to 89)% (area under the ROC curve, 0.564 (0.398 to 0.720), *P *= 0.48 vs. 0.500 and *P *< 0.05 vs. the AUC for CIpc; Table 3, Figure 2). After volume expansion, the bias between the absolute values of CIpw and CItd was -0.32 ± 1.03 L/min/m^2^, and the percentage error was 58%.

### Comparisons of CIpc and CIpw with CItd in Group 2 (introduction/increase of norepinephrine)

In Group 2, the introduction or increase of norepinephrine significantly increased the mean arterial pressure, CItd, CIpc, CIpw, and the SVR by 21 (5 to 36)%, 13 (7 to 22)%, 10 (3 to 17)%, 8 (0 to 18)% and 19 (9 to 31)%, respectively (Table 2).

**Figure 3:**
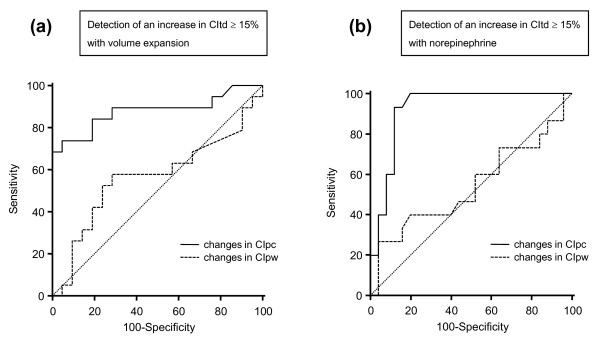
**Bland-Altman plots**. **(a, b) **Bland-Altman plot for the changes (in absolute values) and correlation (for the percentage changes) induced by *the introduction/increase in norepinephrine *on cardiac index obtained by transpulmonary thermodilution (CItd) and cardiac index obtained by the pulse-contour analysis (CIpc). **(c, d) **Bland-Altman plot for the changes (in absolute values) and correlation (for the percentage changes) induced by *the introduction/increase in norepinephrine *on cardiac index obtained by transpulmonary thermodilution (CItd) and cardiac index obtained by the arterial-pressure waveform analysis (CIpw). Plots: straight line, bias; dashed line: +2SD/-2SD limits of agreement); dashed line, correlation line.

The bias between the absolute changes in CIpc and CItd induced by norepinephrine introduction/increase was 0.05 ± 0.74 L/min/m^2^. The coefficient of correlation between the norepinephrine-induced percentage changes in CIpc and in CItd was 0.78 (*P *< 0.05) (Table 3, Figure 3). An increase in CIpc ≥ 15% detected an increase in CItd induced by volume expansion with a sensitivity of 93 (68 to 99)% and a specificity of 88 (69 to 97)% (area under the ROC curve, 0.924 (0.795 to 0.983), *P *< 0.05 vs. 0.500; Table 3, Figure 2). After the introduction/increase of norepinephrine, the bias between the absolute values of CIpc and CItd was 0.05 ± 0.36 L/min/m^2^, and the percentage error was 23%.

The bias between the absolute changes in CIpw and CItd induced by norepinephrine introduction/increase was -0.01 ± 1.75 L/min/m^2^. The coefficient of correlation between the norepinephrine-induced percentage changes in CIpw and in CItd was -0.003 (*P *= 0.65) (Table 3, Figure 3). An increase in CIpw ≥ 34% detected an increase in CItd induced by volume expansion with an sensitivity of 27 (8 to 55)% and a specificity 96 (80 to 99)% (area under the ROC curve, 0.541 (0.377 to 0.700), *P *= 0.66 vs. 0.500 and *P *< 0.05 vs. the AUC for CIpc; Table 3, Figure 2). After the introduction/increase of norepinephrine, the bias between the absolute values of CIpw and CItd was 0.01 ± 0.94 L/min/m^2^, and the percentage error was 60%.

### Effects of changes in SVR on the agreement of CIpc and CIpw with CItd

Considering the whole population, the SVR increased by 5 (-10 to 19)%. The bias between the changes in CIpc and CItd was not significantly correlated with the changes in SVR. By contrast, the bias between the changes in CIpc and CItd was significantly correlated with the changes in SVR (*r *= 0.43; *P *< 0.05).

In the subset of patients in whom the SVR changed by <15% (n = 36), the bias between the absolute changes in CIpc and CItd was -0.07 ± 0.63 L/min/m^2^, and the bias between the absolute changes in CIpw and CItd was -0.21 ± 1.01 L/min/m^2^. The coefficient of correlation between the percentage changes in CItd and CIpc was 0.64 (*P *< 0.05), and the coefficient of correlation between the percentage changes in CItd and CIpw was 0.47 (*P *< 0.05).

In the subset of patients in whom the SVR increased by >15% (n = 44), the bias between the absolute changes in CIpc and CItd was -0.08 ± 0.70 L/min/m^2^, and the bias between the absolute changes in CIpw and CItd was -0.06 ± 1.79 L/min/m^2^. The coefficient of correlation between the percentage changes in CItd and CIpc was 0.78 (*P *< 0.05), and the coefficient of correlation between the percentage changes in CItd and CIpw was -0.15 (*P *< 0.05).

### Effects of connecting the Vigileo device to the femoral or the radial arterial lines

Considering the 20 patients in whom the Vigileo was successively connected to the radial and the femoral arterial lines before and after the therapeutic intervention (40 pairs of measurements), the CIpw values measured from the radial and the femoral lines were not statistically different (2.9 (2.6 to 3.1) vs. 2.8 (2.6 to 3.1) L/min/m^2^; bias, -0.08 ± 0.40 L/min/m^2^). In these patients after the therapeutic intervention, the bias between the absolute values of CIpc and CItd was -0.11 ± 0.22 L/min/m^2^, and the percentage error was 14%. After the therapeutic intervention, the bias between the absolute values of CIpw connected to the radial line and CItd was -0.47 ± 0.84 L/min/m^2^, and the percentage error was 57%.

### Effects of the mode of connection of the PiCCO and Vigileo devices on the femoral arterial line

Considering the 20 patients in whom the femoral arterial line was alternatively directed toward the sole PiCCO or Vigileo devices or directed in a Y to both, before and after therapeutic interventions (40 pairs of measurements), the CIpc was not different when the femoral arterial line was connected only to the PiCCO device or when it was directed in a Y to the PiCCO and the Vigileo devices (3.1 (2.6 to 3.8) vs. 3.0 (2.8 to 3.9) L/min/m^2^; bias, 0.02 ± 0.31 L/min/m^2^). The CIpw was not different when the femoral arterial line was connected only to the PiCCO device or when it was directed in a Y to the PiCCO and the Vigileo devices (2.9 (2.6 to 3.2) vs. 2.9 (2.6 to 3.2) L/min/m^2^; bias, 0.04 ± 0.38 L/min/m^2^).

### Variation of CItd, CIpc, and CIpw

The coefficient of variation was 6.8% for the CItd, 1.8% for CIpc, and 2.0% for CIpw.

## Discussion

We have shown that the calibrated pulse contour-derived CI accurately tracked the changes in CI induced by volume expansion and norepinephrine in sepsis patients. By contrast, the uncalibrated arterial pressure waveform-based CI tracked the changes in CI induced by those therapeutic interventions with less accuracy. The more the SVR changed, the less the uncalibrated arterial-pressure waveform analysis was accurate for monitoring the changes in CI.

### Difference of the devices for the measurement of arterial waveform-derived cardiac output

Among the techniques that have been developed as an alternative to the pulmonary artery catheter, transpulmonary thermodilution has been demonstrated to be reliable for measuring cardiac output as compared with classic thermodilution [3-6,8,9] and we used it as a reference in the present work.

Different commercial devices use the arterial-pressure waveform analysis for providing a real-time estimation of stroke volume and cardiac output. The PiCCO device calculates stroke volume by measuring the area under the systolic portion of the arterial-pressure curve and dividing it by the aortic impedance. The latter is determined by calibration against a measure of cardiac output by transpulmonary thermodilution. In addition, this device enables tracking the changes in arterial compliance. At the time of calibration, arterial compliance is calculated from the time constant of the pressure decay in diastole (t) and SVR (compliance t/SVR). Then, compliance and resistance are updated from beat to beat, according to a proprietary algorithm that depends particularly on the arterial pressure (P) and on dP/dt. A specific patient-calibration factor (cal) (which is independent of compliance and resistance) is added to the formula, which computes the pulse-contour cardiac output [7]. This estimation of cardiac output from the pulse-contour analysis has been demonstrated as reliable by numerous studies in various clinical settings [4-8,13,32,34-37].

The Vigileo device differs from the PiCCO device in two main points. First, it does not take into account the area under the systolic part of the arterial curve, but does the standard deviation of the points contained by the arterial curve in a beat. Second, it does not determine aortic impedance from any external calibration of cardiac output, but estimates it from pressure-waveform characteristics, such as skewness and kurtosis, and from patient demographic data (age, gender, height, and weight) [10]. With the software we used in the present study, the estimation of arterial compliance is updated on a rolling 60-second average. Whereas some studies demonstrated an acceptable agreement between CIpw and CI measured either by pulmonary or transpulmonary thermodilution [11-15,37], other studies reported conflicting results [17,18,20,21,24]. In particular, it has been suspected that CIpw could be inappropriate for estimating CI in the case of low SVR [20,24] or when the arterial waveform changes to a large extent [17]. The most recently commercial version of the device demonstrated a poor ability to measure CI in critically ill patients with hemodynamic instability [16].

### Accuracy of the two arterial-pressure waveform analyses for tracking changes in cardiac output

In the present study, the CIpc showed good accuracy in tracking the CItd changes induced by the therapeutic interventions. Moreover, it was able to detect correctly the increases in CItd of ≥15% in ROC curve analysis. Interestingly, this ability was not altered by the level of the changes in SVR, confirming what we recently reported [32]. It is noteworthy that we used the most recent version of the device that has been improved for tracking the changes in arterial impedance.

By contrast, in our sepsis patients, the uncalibrated estimation of CI poorly tracked the short-term changes in CItd during volume expansion and in those secondary to norepinephrine introduction/increase. Furthermore, the CIpw was unable detect correctly the changes in CItd ≥ 15%, induced either by volume expansion or by norepinephrine introduction/increase. The precision of CIpw device was high (that is, the values of repeated CIpw measurements were close) [38], but its accuracy compared with that of CItd was low. We could not further investigate the technical limitation that made the uncalibrated estimation of CI inaccurate, because we could not analyze all the components of the proprietary algorithm. Nevertheless, we found that the low accuracy of CIpw was related to the magnitude of SVR changes: the more the SVR changed, the higher the bias between the changes in CIpw and CItd. This suggests that the limitation of the system probably resides in an incorrect estimation of the resistive component of the cardiovascular system. It must be acknowledged that when the SVR did not change to a large extent, the reliability of CIpw was acceptable and in agreement with recent studies conducted in nonsepsis patients [13,15,19,37]. By demonstrating that the CIpw was identical when measured from the radial and femoral arteries, we ruled out the possibility that the inaccuracy of CIpw was related to the fact that the device was more frequently connected to the femoral artery. Also, by checking that the CIpc and CIpw were identical when the femoral arterial line was connected to only one of the devices or when it was split in a Y between the two devices, we ruled out the influence of some damping phenomenon of the arterial line on the results observed in the whole population.

### Limitations

First, the CIpc was calibrated at baseline such that before therapeutic interventions, the CIpc was similar to the CItd. In this regard, it could be considered that the CIpc was advantaged compared with the CIpw. Nevertheless, the calibration of the CIpc was performed only at baseline and thus could not affect the comparison of the ability of CIpc and CIpw to track changes in CI induced by the therapies.

Second, we did not use the pulmonary artery thermodilution as a reference. Nevertheless, the accuracy of the transpulmonary thermodilution to measure cardiac output has been repeatedly demonstrated [4-6,8,13,32,34-37].

Third, we investigated the reliability of CIpc and CIpw to track short-term changes in CI. If tested during a longer calibration-free period (for example, >1 hour), the reliability of CIpc could be less. Finally, we used the data that were automatically displayed by both devices (that is, after processing, filtering, and averaging). Thus, we could not precisely analyze the reason that one device performed differently. The filtering and averaging of data by the devices also likely explained part of the low variability of CIpc and CIpw.

## Conclusions

The pulse contour-derived estimation of CI provided was accurate for assessing the changes in CI induced by volume expansion and norepinephrine in sepsis patients. By contrast, the uncalibrated estimation of CI by arterial-pressure waveform analysis was not of sufficient accuracy for monitoring CI changes in this setting. Whether a new generation of the Vigileo device would perform better than the current commercial model we used remains to be demonstrated.

## Key messages

• In septic shock patients, the pulse contour-derived estimation of cardiac index correctly tracked the changes in cardiac index induced by volume expansion and norepinephrine.

• The pulse-wave analysis was less reliable for tracking the changes in cardiac index induced by those therapeutic interventions.

• The ability of the pulse-wave analysis to track the changes in cardiac index was poorer when the systemic vascular resistance changed to a large extent.

• Whether more-recent versions of the devices could perform differently should be assessed in further studies.

## Abbreviations

CI: cardiac index; CItd: CI measured by transpulmonary thermodilution; CIpc: CI measured by the PiCCO pulse-contour analysis; CIpw: CI measured by FloTrac/Vigileo pressure-waveform analysis; SVR: systemic vascular resistance.

## Competing interests

Professors Jean-Louis Teboul and Xavier Monnet are members of the Medical Advisory Board of Pulsion Medical Systems. The other authors have no financial competing interest to disclose.

## Authors' contributions

XM conceived the study, contributed to the collection of data, performed analysis and interpretation of the data, and drafted the manuscript. NA contributed to the collection of data. BN contributed to the collection of data. JJ contributed to the collection of data. CR participated in the coordination of the study and helped to draft the manuscript. J-LT conceived the study, coordinated the collection, analysis, and interpretation of the data, and helped to draft the manuscript. All authors read and approved the final manuscript.
